# Beyond Trust: Plagiarism and Truth

**DOI:** 10.1007/s11673-017-9825-6

**Published:** 2017-12-12

**Authors:** Bart Penders

**Affiliations:** Care and Public Health Research Institute (Caphri), Department of Health, Ethics and Society (HES), Maastricht University, PO Box 616, NL-6200MD Maastricht, The Netherlands

**Keywords:** Trust, Truth, Plagiarism, Misconduct, Responsible research behaviour, Epistemology

## Abstract

Academic misconduct distorts the relationship between scientific practice and the knowledge it produces. The relationship between science and the knowledge it produces is, however, not something universally agreed upon. In this paper I will critically discuss the moral status of an act of research misconduct, namely plagiarism, in the context of different epistemological positions. While from a positivist view of science, plagiarism only influences trust in science but not the content of the scientific corpus, from a constructivist point of view both are at stake. Consequently, I argue that discussions of research misconduct and responsible research ought to be explicitly informed by the authors’ views on the relationship between science and the knowledge it produces.

Scientific research practices and the scientific life are increasingly object of critical scrutiny. Evaluation cultures and audit practices are on the rise and are shaping scientific behaviour (Rushforth and de Rijcke, 2015). Of course, those of us using public resources to pursue research should be able to account for what we do, to our publics and our peers. Akin to the growing evaluation and audit of scientific practices, attention to research integrity and responsible research is on the rise. These trends meet one another in the discourse on, for instance, research waste, yet oppose one another which it comes to, for instance, critical discussions on research metrics. The growing body of scholarly work dealing with research integrity has historically been describing and analysing cases of misconduct—large and small. Only more recently has this been seriously supplemented with work on fostering responsible research or detailed studies of questionable research practices. What is absent from nearly all of these studies are explicit connections between the normative framework for assessing or studying research integrity on the one hand and different views on how the sciences make knowledge on the other. If the latter vary, what does this mean for our views on research integrity? I will demonstrate this via a critical analysis of the status of plagiarism as fraud.

Plagiarism is listed among the three deadly sins in science along with fabrication and falsification in most international literature on research integrity. However, it is often demoted to a second rank in the annals of fraud, since it is argued that plagiarism does not corrupt the content of science, only the distribution of credit in it, whereas fabrication and falsification do both. Or, in different words, it is not hindering science on its relentless quest for truth, while fabrication and falsification do. Plagiarism is, according to this view, still wrong and still an act of fraud and scientific misconduct, but it has little to no consequence for science’s problem-solving ability or truth-making ability (Goodstein 1995). Of course, it still significantly influences public perceptions of scientists, since it suggests that scientists are dishonest and participate in acts such as theft. As an immediate consequence, plagiarism is an important risk for public trust in science in general. True to this perspective, Bouter and colleagues even worked towards quantifying the effect of plagiarism on truth (relatively small) and trust (bigger) (Bouter et al. 2016).

In this critical perspective, I will argue that plagiarism is both an issue of trust and of truth. I will argue that plagarism distorts science’s infrastructure for knowledge making and its capacity for truth-making. Of course, there are existing critiques claiming that plagiarism costs resources to deal with, diminishing their availability for truth-making (Biagioli 2012) or that loss of public trust may translate in a loss of public support for science—including financial and political support—both (only) *indirectly* affecting scientific potential for truth-making. The claim that plagiarism does not directly affect the truth(s) that science generates is based upon a very specific understanding of science—one of an empirical, objective practice of moral professionals who adhere to strict and shared methodological and conceptual guidelines in order to produce pure and neutral knowledge with as little distraction (or bias) as possible.

Based upon this view, who claimed what does not matter for the content of science. How the claim comes into being, does not matter for the total body of knowledge. When it came into being and who claimed it first, are irrelevant. What matters is how the claim is justified (tested, verified, challenged, supported, etc.), not how it came into being. Despite it still being the dominant view of science by the public and practitioners alike, this view of science—in particular the relationship between science and truth—is increasingly contested. The character of truth, the way it is framed and contested, are at the centre of current public debates labelled “post-fact” or “post-truth” (Makri 2017). The role of science and its relationship to truth are at the core of these debates. This relationship between science and truth has been the object of critical scrutiny in the philosophy of science for a very long time. For a number of decades, others have joined in the conversation, most notably the sociology and anthropology of science—coining new understandings of the relationship between science and the knowledge it produces, as well as alternative views of how that knowledge acquires increasing credibility and ultimately the status of truth. Here, I choose the sociology of science and in particular the sociology of scientific knowledge as one such alternative view of science that, as I argue, translates into an alternative view on the fraudulent status of plagiarism.

The sociology of science asks why groups of scientists agree on such things as the boundaries of science (Gieryn 1983, 1999); why they choose to work within the confinements of specific paradigms (Kuhn 1970 [1962]), ways of knowing (Pickstone 2000), thought styles (Fleck 1980 [1935]), styles of reasoning or thinking (Crombie 1994; Hacking, 1992a, 1992b, 2002), and why such epistemic cultures or thought collectives exist in the first place (Fleck 1980 [1935], Knorr-Cetina 1999). It is the social and epistemic organization of the sciences and the ways in which they influence one another that motivates sociologists in their study of science (Hackett et al. 2016). Starting with the works of Ludwik Fleck in the 1930s and more prominently with the work of Thomas Kuhn in the 1960s and 1970s, sociological objects, such as shared beliefs and belief systems, reward and credit structures in science, processes of socialization into science and sense of community—to name just a few—moved into the heart of epistemology, forever changing the way we look at the relationship between science and the knowledge it makes.

Of course, the sociology of scientific knowledge (SSK) is not a fully homogenous practice and it is closely intertwined with the history and anthropology of science and science and technology studies (STS). If we take a look at one of the most “radical” epistemic positions in SSK, the relevance of taking such positions into account in debates on research integrity becomes most clear. I am talking about the “strong programme” in the sociology of science, which states that sociology is strong in the sense that it can help us understand how both “true” and “false” theories emerge as such in science (as opposed to a weak sociology which can only help us understand why failed theories failed). The strong programme does so through organizing its view on science through four distinct characteristics: it studies causality and the social or cultural conditions that help generate claims to begin with; it is impartial to the truth in the sense that it studies unsuccessful and successful claims; it is symmetrical in its study of claims and knowledge though using the same or similar explanations to account for success or failure and finally it is reflexive in the sense that all of the above will have to apply to sociology itself as much as to the science it studies (Bloor 1991 [1976]).

The path to consensus about claims thus becomes the object of study. Whether that path leads to a consensus that the claim is nonsense or a consensus that the claim is an irrefutable truth, does not change the way in which one ought to study that path. This is an example of social constructivism, an epistemic position that puts credibility and consensus ahead of truth: only with enough credibility and a consensus that is shared widely enough and by the right actors, will a theory or claim acquire the status of truth or fact. In the sociological study of how consensus arises and how credibility is gathered, a lot of ingredients begin to matter that did not matter beforehand: who came up with a claim or theory? What is this person’s status? That status can be about the employing institution (Harvard and Oxford have a lot, one’s local community college has little) or an individual’s track record (credibility built among peers with publications, grants, (Nobel) prizes and more). It can be about the rhetorical strategies employed in texts or elsewhere, social ties between institutions and individuals that existed before the claim was ever coined. The study of consensus-building is also about power distributions: one who has little power cannot build international consensus by herself—powerful and strong allies with international reputation and prestige are required to lift the status or credibility of a claim. Consensus is social and political and as a consequence, so is science and its claims.

If we view science as a practice in which consensus matters for establishing claims as true, the route towards that consensus will determine whether or not a truth is ever established as such. Who acts in or is involved willingly or unwillingly in this consensus-forming culture, is of enormous influence on which consensus will ultimately be established—internalizing the many interests that shape science—careers, ideology, resources, and many more (Bero and Grundy 2016). The social and political dynamic that is consensus formation is not blind to the origin of a claim or the support it gathers along the way. The origin, as well as the amount and character of that support is obscured through, for instance, acts of plagiarism.

To plagiarize in the context of the epistemic position held by SSK amounts to illegitimately claiming credit, reputation, and prestige to assist consensus-building. If credibility and reputation are the basis of science—as, amongst others, Randall Collins (1975) has helped establish—this means that the path to consensus and potentially the route towards establishing a theory or claim as true, lacks the support its boasts. As a result, in the epistemic position as held by SSK, plagiarism is not just a risk for the trust placed by peers or the public in science in general or scientists in particular. Far more, it pulls out the rug from underneath the consensus that establishes truth and is fully equivalent to fabrication or falsification as a scientific transgression.

However, plagiarism is not the only scientific misconduct or questionable research practice that targets the allocation of credit and credibility. Others include authorship disputes, most notably the inclusion of prominent scientists, designed to help build credibility for a paper and a claim, or citation rings, artificially boosting the recognition and prestige of a few publications (and thus a select set of claims) over competing ones. Of course, it can be very hard to decide who is or is not a legitimate author, despite the existence of guidelines and various local implicit norms (Penders 2016, 2017). The same goes for what counts as “proper” citation. However, that does not excuse “credibility-fraud” and the risk it poses to both the public and professional status of science and the epistemic processes inside science (Martin 1994).

Sociology of scientific knowledge and the strong programme are not the only epistemic positions that differ from dominant positivist views of science. Each comes with an understanding of what counts as normal behaviour—individually, culturally, and (infra)structurally—and interpretations of what deviance from that normal means. Published and unpublished positions on research integrity and responsible research practices do not, generally, disclose the epistemic position from which they originate. Of course, the strong programme is a radical epistemic position in its restriction to social determinants for truth or falsehood via the route of consensus formation. However, other theories of knowledge production move away from the purely social and incorporate social and material or physical elements in their foundations for truth—including quite prominently, Actor-Network Theory (Latour and Woolgar 1979). These constructivist theories rely on network-formation, allies (both social and physical), and more, to explain where knowledge and its status comes from. To them, the origin of a claim, the ways in which it is translated, and who participates in that translation matter to the point that plagiarism and authorship disputes directly influence what can and what cannot ultimately become accepted, credible, and true.

The scientific community at large does not share a single epistemic position. This translates into the coexistence of various positions on how science makes knowledge in practice but also on how this is to be done ideally. Similarly will the severity of digressions be evaluated differently—as argued above in the case of plagiarism—to the point that disagreement may exist on a number of questionable research practices and whether or not they qualify as questionable research practices to begin with. In order to help understand these differences, our studies of plagiarism and other acts or practices of misconduct and questionable research practices on the one hand, and research integrity and responsible research practices on the other, best heed the epistemic position of research, researchers, and claims. This is not to allow excuses or exceptions but to be able to pass fair judgement (Penders, Vos, and Horstman 2009). The same goes for our teaching of research integrity and responsible research (Grinnell 2013). This will require conceptual and empirical studies of responsible research—and of questionable research—symmetrically, impartially, and with a focus on the relationship between notions of science in practice and proper science.
